# A Novel Dual-Language Touch-Screen Intervention to Slow Down Cognitive Decline in Older Adults: A Randomized Controlled Trial

**DOI:** 10.1093/geroni/igae052

**Published:** 2024-05-27

**Authors:** Wei Quin Yow, Ka Lon Sou, Alina Clarise Wong

**Affiliations:** Humanities, Arts, and Social Sciences, Singapore University of Technology and Design, Singapore, Singapore; Humanities, Arts, and Social Sciences, Singapore University of Technology and Design, Singapore, Singapore; Humanities, Arts, and Social Sciences, Singapore University of Technology and Design, Singapore, Singapore

**Keywords:** Bilingualism, Cognitive intervention, Cognitively impaired, Dementia, Verbal memory

## Abstract

**Background and Objectives:**

Bilingualism has been suggested to protect older adults from cognitive aging and delay the onset of dementia. However, no studies have systematically explored bilingual usage as a tool to mitigate age-related cognitive decline. We developed the Dual-Language Intervention in Semantic memory—Computerized (DISC), a novel cognitive training program with three training tasks (object categorization, verbal fluency, and utility of things) designed specifically for older adults that featured two modes: single-language (SL) exposure mode and dual-language (DL) exposure mode.

**Research Design and Methods:**

The final sample included 50 cognitively healthy (CH; 33 female, *M*_age_ = 72.93 years, range = 53.08–87.43 years) and 48 cognitively impaired (CI; 35 female, *M*_age_ = 80.93 years, range = 62.31–96.67 years) older adults, randomly assigned them into one of three groups: SL group, DL group, and control group (no training). Participants in SL and DL groups used DISC in either SL mode (i.e., training instructions were spoken in only one language throughout the entire training) or DL mode (i.e., training instructions alternated between two languages), respectively, for 24 sessions. Participants in the control group were asked to continue with their normal daily activities (e.g., playing bingo and reading newspapers).

**Results:**

For CH older adults, we found significant improvements in the Rey Auditory Verbal Learning Test (RAVLT) Trial 5 score and the Clock Drawing Test score in the DL group but not in the SL and control groups posttraining compared with pretraining. For CI older adults, there was a delayed improvement in the RAVLT Trial 1, six months later.

**Discussion and Implications:**

Our findings provided novel evidence that implementing DL cognitive training benefits CH older adult’s late verbal learning and visuospatial construction skills, and a delayed improvement in CI older adults’ early verbal learning abilities.

**Translational Significance:** With an aging population, the prevalence of cognitive impairment is growing. However, traditional cognitive training has produced inconsistent benefits in older adults. We implemented the Dual-Language Intervention in Semantic memory—Computerized (DISC) to garner the cognitive benefits associated with bilingualism. Medium to large effects were found in both cognitively healthy and cognitively impaired older adults after dual-language DISC training, including enhanced verbal memory. DISC was also developed with user-friendliness and scalability in mind in order to reach the wider community. Most importantly, our findings support the effectiveness of dual-language use in mitigating cognitive decline, which can be incorporated into existing dementia care and treatment.

## Background and Objectives

Cognitive abilities such as processing speed, memory, verbal fluency, visuospatial construction skills, and executive functioning skills tend to decline in older adults (Deary et al., 2009; Harada et al., 2013). Studies that examined age-related structural and functional changes found that human brain volume decreases by about 0.4% every year, and the degeneration rate, especially in the temporal region, further increases after 70 years old (Fujita et al., 2023). Such age-related neuronal volume loss, especially in white matter, in turn disrupts verbal memory and executive function (e.g., Coelho et al., 2021). Although healthy aging is possible in the midst of cognitive decline (see Park & Reuter-Lorenz, 2009), an acceleration of these neurocognitive alterations could indicate neurological disorders. Deficits in declarative memory (i.e., semantic and episodic memory) and general cognitive function are typical markers of Alzheimer’s disease (AD; Irish et al., 2016; Zhao et al., 2014).

Interestingly, studies have found that lifelong bilingualism may contribute to neuroplasticity and cognitive reserve in a way that helps stave off symptoms of mild cognitive impairment (MCI), dementia, and AD for longer periods of time (e.g., Anderson et al., 2020; Bialystok et al., 2014; Costumero et al., 2020; Perani et al., 2017; however, see Yeung et al. (2014); the discrepancy could stem from conflating incidence rates of dementia and the age at which the symptoms first appear, as well as statistical and methodological issues in the study designs). More recently, Brini et al. (2020) conducted a systematic review and meta-analysis and found that, across 21 studies, bilingualism delays the onset of symptoms of AD by an average of 4.7 years and diagnosis with dementia by an average of 3.3 years. While several longitudinal prospective studies did not find a risk reduction in developing dementia among bilinguals relative to monolinguals (e.g., Ljungberg et al., 2016), other studies found that learning a foreign language in childhood and adolescence is associated with a lower risk of developing MCI in old age (e.g., Wilson et al., 2015).

Similarly, bilingual older adults have been found to have enhanced cognitive control compared with monolingual older adults due to a greater need for bilinguals to suppress interfering cues and resolve cross-linguistic competition (e.g., Bialystok & Craik, 2010; Freeman et al., 2017; Green & Abutalebi, 2013), such as transient control and goal maintenance during task-switching (e.g., López Zunini et al., 2019). A meta-analysis study conducted by Chen and colleagues (2022) concluded that there is a small but significant bilingual advantage in executive function in older adults, particularly in older adults with MCI. However, several studies have reported no bilingual benefits or even bilingual disadvantages in older adults’ executive function (e.g., Antón et al., 2016; De Bruin et al., 2015). Degirmenci et al. (2022)’s systematic review reported no evidence for the cognitive benefits of bilingualism on healthy older adults. Several factors could explain the discrepancies between these studies. First, most of the studies measured bilingualism via self-reported questionnaires. The accuracy of the profile may then be confounded by the participant’s memory and be susceptible to misreporting. Second, there is much variability in bilingual experiences across people and cultures, and there is no consensus on one “best” way to define and measure bilingualism. It is suggested that researchers should take into account the local bilingual context, practices, and educational background when measuring bilingualism (Kremin & Byers-Heinlein, 2021; Surrain & Luk, 2019).

One way to test the effects of bilingualism on cognitive functioning in older adults while addressing the earlier limitations is to focus on one aspect of bilingualism, for example, bilingual usage, and then systematically vary that language variable. Past studies have suggested that learning a foreign language can help enhance cognitive abilities in children and protect against cognitive decline in healthy older adults (e.g., Antoniou et al., 2013; Kroll & Dussias, 2017). Bak (2016) recruited a group of older adults to participate in an intensive language course and found that participants who completed the language course were better at attention-switching than those who did a course on something else. A review study by Klimova (2018) suggested that learning a foreign language may offer benefits to older adults via enhancement of cognitive functioning. However, adopting foreign language learning as a cognitive intervention in the community for older adults has its challenges. For example, older adults might take more time and effort to learn a new language as they have difficulty distinguishing new sounds and retrieving novel words (Klimova, 2018). It might also be difficult to maintain the regular use of the new language in a monolingual population or in a population where the new language is not typically used in everyday life.

Rather than teaching older adults a new language, another way to vary their bilingual usage is to shape the extent of usage of two *known* languages of a bilingual population through an intervention program. According to the adaptive control hypothesis (Green & Abutalebi, 2013), bilingual speakers’ control processes adapt according to the cognitive demands they face during a conversational interaction. In a dual-language (DL) context, where two languages are spoken in one environment (language switching is allowed within a conversation), speakers engage in salience cue detection, response inhibition, task engagement, and task disengagement, which exceed the cognitive demands that are engaged in the single-language (SL) context. Thus, immersing in a DL context could enhance cognitive functioning significantly more than in a SL context. Indeed, studies found that young bilinguals who are more exposed to the DL context have better cognitive flexibility (as indexed by smaller switching cost and mixing cost in a task-switching task) than those who are more exposed to the SL context (Hartanto & Yang, 2016; Khodos & Moskovsky, 2021). Educational programs that support a DL approach, where elementary school children spend a certain ratio of their school time learning school subjects (e.g., Mathematics, and Science) in a foreign language and the rest of the time in their native language, also found that students in DL program performed better on their academic subjects, verbal working memory, verbal learning, and inhibition than their SL counterparts (e.g., Esposito & Baker-Ward, 2013; Kaushanskaya et al., 2014; Neveu et al., 2021; Park et al., 2023). A recent study of bilingual older adults by Chan et al. (2020) in Singapore revealed that active bilingualism, indicated by more balanced bilingual usage, was associated with better performance in goal maintenance in older adults. Thus, it is viable to test the effects of DL context on cognitive functioning in bilingual older adults. To our knowledge, there are currently no studies that explore bilingual usage as a cognitive intervention to mitigate cognitive decline in older adults.

Here, we tested the hypothesis that a greater use of two languages can elicit significant improvements in verbal learning and cognitive flexibility in bilingual older adults. With this objective in mind, we developed a novel DL cognitive tool to investigate whether DL use can provide significant benefits in mitigating cognitive decline in bilingual older adults as part of a larger project, Dual-Language Intervention in Semantic memory—Computerized (DISC). The cognitive tool, DISC, is an older adult-friendly app with adaptive prompts and difficulty levels administered via an avatar to encourage independent play on a multimodal touchscreen tablet for both cognitively healthy older adults and those with mild-to-moderate dementia.

## Research Design and Methods

### Participants

Fifty-seven cognitively healthy (CH) and 48 cognitively impaired (CI) bilingual older adults were recruited from senior day care centers. Older adults who were diagnosed with visual and auditory disability were not eligible to participate in the study. The older adults live in Singapore, a multilingual and multicultural country with the largest ethnic group being Chinese (about 74% of the total population). Within the Chinese community, older adults are generally knowledgeable in at least two regional Chinese varieties or dialects, e.g., Mandarin, Hokkien, Cantonese, Hakka, Teochew, etc. (Lyu et al., 2015; Sumartono & Tan, 2018). It is common to encounter people engaging in dual language use across various interactional contexts in Singapore. The majority of the older adults in Singapore are bilingual speakers but they vary substantially in the individual usage of the languages (from predominantly one language to a balanced use of two languages; see Chan et al., 2020; Hartanto & Yang, 2020; Yow & Li, 2015). Thus for Singaporean older adults, the extent of usage of these languages becomes an important variable in their language experience that we seek to vary in this study.

Participants’ cognitive state, that is, CI or CH, was reported by the centers. The general inclusion criteria for both groups were (a) aged 40 and above, and (b) the ability to speak two of the following languages: English, Mandarin, Cantonese, and Hokkien. Seven CH participants were excluded as their Mini-Mental State Examination (MMSE) scores were equal to or below 19 (Chua et al., 2019; see Author Note 1). The CI participants were required to have mild to moderate dementia. The final sample consisted of 50 CH (33 female, *M*_age_ = 72.94 years, range = 53.08–87.43 years) and 48 CI older adults (35 female, *M*_*age*_ = 80.93 years, range = 62.31–96.67 years). The four types of dementia present in the CI participants were Alzheimer’s disease, vascular dementia, frontotemporal dementia, and mixed dementia. Table 1 summarizes participants’ demographic information. No significant differences were found between the three groups (DL, SL, and control) in all demographic information in both the CH and CI participants (based on ANOVA and Chi-square test for continuous variables and categorical variables respectively).

**Table 1.** Demographics of Participants with Cognitively Healthy and Cognitively Impaired Older Adults by Group

Variable	Group		
	Dual language	Single language	Control
*Cognitively healthy older adults*			
*N*	18	14	18
Female, *n*	13	10	10
Age (in years)	73.40(8.81)	70.54(8.04)	74.34(7.60)
Years of education	9.94(5.65)	10.21(4.53)	10.61(4.78)
Monthly household Income, *n*			
< S$2,000	12	10	9
S$2,000—S$2,999	2	2	1
S$3,000—S$4,999	4	1	4
> S$5,000	0	1	4
Number of known languages	3.88(0.72)	3.77(0.83)	3.56(0.70)
Highest language proficiency ^a^	4.81(0.36)	4.90(0.28)	4.82(0.34)
Second highest language proficiency ^a^	4.06(0.90)	3.96(0.78)	4.28(0.83)
Highest language usage ^b^	67.0(19.5)	64.3(15.0)	67.8(22.2)
Second highest language usage ^b^	24.7(14.4)	26.4(14.5)	21.9(14.1)
*Cognitively impaired older adults*			
*N*	15	15	18
Female, *n*	11	11	13
Age (in years)	82.94(7.57)	82.81(8.53)	77.69(5.95)
Years of education	7.07(6.32)	4.13(4.73)	5.33(3.51)
Monthly household income, *n*			
< S$2,000	13	12	9
S$2,000—S$2,999	1	1	2
S$3,000—S$4,999	1	2	7
> S$5,000	0	0	0
Number of known languages	3.33(0.82)	3.33(0.82)	2.83(0.71)
Highest language proficiency ^a^	4.87(0.35)	4.80(0.56)	4.76(0.46)
Second highest language proficiency ^a^	3.58(0.73)	4.05(1.13)	3.51(0.97)
Highest language usage ^b^	72.1(12.3)	62.8(14.8)	71.4(18.2)
Second highest language usage ^b^	22.3(11.1)	26.5(10.7)	22.7(13.9)

*Notes*: Standard deviations are presented in parentheses.

^a^Language proficiency was calculated by averaging the proficiencies of listening, speaking, reading, and writing of that language; scores range from 1 to 5.

^b^Language usage was a weighted average of the percentages of the usage time of that language with family members, with colleagues, with friends, and in other contexts based on the percentage of time spent in each of the four contexts; scores range from 0 – 100.

### Intervention Program

**Figure 1.**
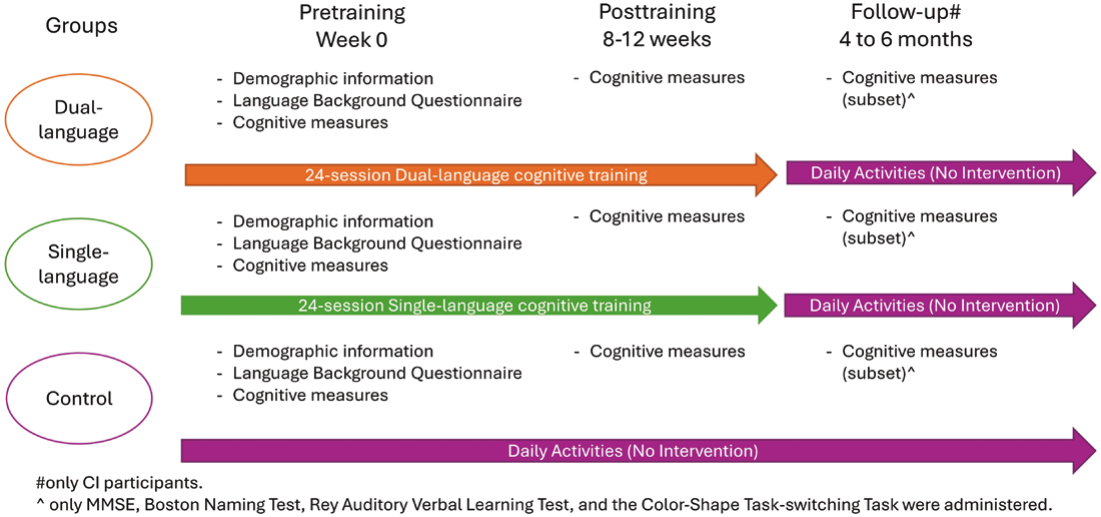
Flowchart depicting the procedure of the study data collection. CI = cognitively impaired; MMSE = Mini-Mental State Examination.

The intervention program included a total of 24 training sessions, which were spread across 8-12 weeks (about 2–3 sessions a week; see Figure 1). The data was collected between July 2019 to November 2021. DISC was administered on a touchscreen computer and headphones with built-in microphones were provided for participants to complete the session. Each session comprised three game tasks (Object Categorization, Verbal Fluency, and Utility of Things), and each task included a baseline measure, two practice trials, and six actual trials (for summary, see Supplementary Material Section A). Each session lasted about 20–40 min. Prior to the COVID-19 outbreak, at least one researcher monitored 3–4 participants at the same time. During the COVID-19 outbreak, to meet the COVID-19 health care restrictions, the sessions were carried out over Zoom with one researcher watching 1–2 participants concurrently. After the restrictions were eased, in-person sessions resumed.

### Game Design

**Figure 2.**
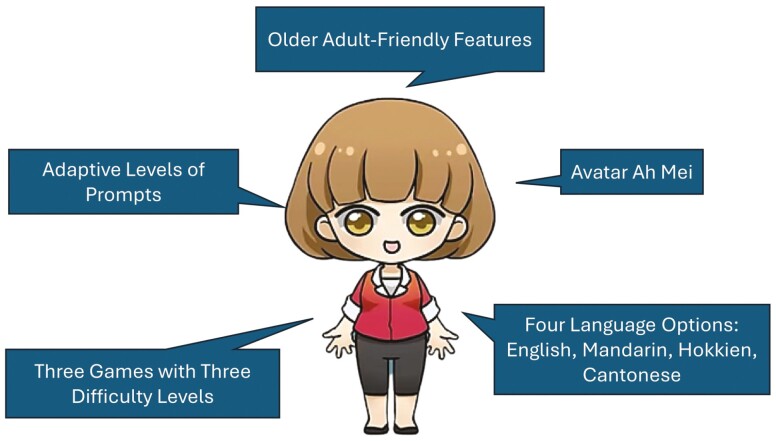
Key features of DISC cognitive training program. DISC = Dual-Language Intervention in Semantic memory—Computerized.

The key features of DISC (see Author Note 2) are presented in Figure 2 and its design summary is presented in Supplementary Material Section B. Three game tasks are developed based on past studies that used similar intervention tasks (Brewer et al., 1981; and Flicker et al., 1987 for the Object Categorization task; Monsch et al., 1992 for the Verbal Fluency task; Tárraga et al., 2006 for the Utility of Things task) and administered through a touchscreen device. DISC features an avatar called Ah Mei (a familiar local female name in Singapore), modeled after a senior care center staff, that delivers instructions, feedback upon completion of tasks, and prompts if participants face any difficulties while playing the game. Instructions use colloquial phrases for ease of understanding and are delivered in a positive tone. Participants can touch the avatar for help where needed. With each touch, the avatar would voice prompts of four different levels, with prompt 1 giving the simplest level of help (e.g., repeating the instructions) and prompt 4 giving the most help (e.g., removing all incorrect options).

There are two language modes available in DISC—SL mode and DL mode. The user can choose to play DISC in one or a combination of two out of four, language options: English, Mandarin, Cantonese, and Hokkien, which are commonly used languages among older adults in Singapore. In the SL mode, all task instructions are delivered in one language. In the DL mode, the task instructions alternate between two languages in a single trial. The design of the DL mode is modeled after the DL context in bilingual communication, which is expected to render the most cognitive demands as per the adaptive control hypothesis (Green & Abutalebi, 2013).

### Pretraining and Posttraining Measures

All pretraining and posttraining sessions’ were administered in one of the four languages (i.e., English, Mandarin, Cantonese, and Hokkien) according to the participants’ dominant language. Both pretraining and posttraining sessions were conducted over two sessions each (i.e., Session A and Session B). Session A includes the Digit Symbol Substitution Test (DSST), the Rey Auditory Verbal Learning Test (RAVLT), the Clock Drawing Test, and the Boston Naming Test, and Session B includes the -MMSE, Color-Shape Task-Switching Task, and N-back Task. The order of the tasks within the testing session was randomized. In addition, a demographic questionnaire and a language background questionnaire were administered during the pretraining sessions.

#### Language Background Questionnaire

The Language Background Questionnaire (LBQ; adapted from Yow & Li, 2015) consisted of questions related to the participant’s daily usage of each of the languages they know and their proficiency in these languages. The participants were asked the amount of time (in percentage) they used each of the languages in different contexts (e.g., home, work, and school) in a week. Usage of a language is the weighted sum of its usage in various contexts based on the time they spent in these contexts. For proficiency scores, participants were asked to rate their proficiency in writing, reading, listening, and speaking in each of the languages they know (5-point scale; 1 is *not proficient* and 5 is *very proficient*). The overall proficiency of a language is the average of the four proficiency subscores. Overall, there were no significant differences in the language profile between participants of the three conditions (control, SL, and DL).

#### Cognitive measures

The participants completed the MMSE, RAVLT Test, and Clock Drawing Test. The participants also completed the Boston Naming Test, DSST, Color-Shape Task-Switching Task, and N-back Task, but as no significant differences in pre–post effects between the three groups were found, we only describe these four tests in Supplementary Material Section C.

##### MMSE

We used the adapted version of MMSE for Singapore participants by Feng et al. (2012) to assess the participants’ cognitive state (Folstein et al., 1975). CH participants were excluded if their education-adjusted MMSE scores did not meet the cutoff scores for multiethnic Asian populations, that is, equal to or below 19 for primary school and below 23 for secondary school graduates (Chua et al., 2019). Based on this criteria, seven CH participants were excluded.

##### RAVLT

We used the Singapore version of RAVLT (Lee et al., 2012). RAVLT is a widely used neuropsychological tool that assesses verbal learning memory. Participants were asked to recall the same list of 15 words immediately after the researcher read out the list five times (Learning Trial 1–5). After that, participants were required to remember and recall a new list of 15 words once. Twenty minutes later, the participants were asked to recall the first list of 15 words again (Delayed Recall Trial). The range of total possible scores was 0–15 per trial.

##### Clock Drawing Test

This is a nonverbal pencil and paper task that measures visuospatial attention, motor skills, conceptualization, and planning. Participants were asked to draw a clock indicating *ten minutes after ten* on a predrawn circle on an A4-sized sheet of paper. The 16-point scoring system was applied (Lin et al., 2003).

#### User Experience and Feedback

We also collected feedback from the staff of the care centers where the study was carried out after the study was completed. The feedback questionnaire contained 15 items, including asking them to rate their satisfaction with DISC (on a Likert scale of 1–5), their extent of agreement on whether DISC can be integrated into their current center programs (on a Likert scale of 1–5), whether they have more time to focus on other clients while the researchers are running the DISC, whether they can perform more duties than they normally did while the researchers were running the DISC, their estimated amount of extra time they could spend on other duties, and other general feedback and suggestions on future updates of the DISC program.

### Procedure

Participants were randomly assigned to either the control group, the SL group, or the DL group after obtaining informed consent. In the pretraining sessions, participants were asked to complete the demographic and language background questionnaires and the cognitive measures. All participants attended the care centers as part of their weekly routine on the training days. Those in the control group were asked to continue with their regular activities supervised by the care center staff, for example, reading newspapers, playing bingo, doing arts and crafts, etc., while those in the SL and DL groups participated in the training sessions. Participants in the SL group were asked to choose the most dominant language that would be used throughout all the sessions in the intervention program. Participants in the DL group were asked to choose their two most dominant languages (out of the four available) as the first and second languages of the training sessions respectively. All participants attended posttraining sessions to complete the same cognitive measures as in pretraining either after completion of 24 training sessions (for the SL group and the DL group) or after approximately 12 weeks (for the control group). As the study spanned across the COVID-19 outbreak period, 38 CH older adults and 23 CI older adults participated in the pretraining sessions in person, while 10 CH older adults and 24 CI older adults participated remotely. On the other hand, 38 CH older adults and 24 CI older adults participated in the posttraining sessions in person, while 11 CH older adults and 24 CI older adults participated remotely. Information on whether sessions were conducted remotely or in-person was missing for two CH and one CI older adults in the pretraining sessions, and for one CH older adults in the posttraining sessions.

A subset of CI participants (*n* = 25) participated in the follow-up assessment six months after the posttraining session to examine the longer-term effect of the intervention on those with dementia. During these six months, the participants did not receive any cognitive training sessions. In the follow-up session, only MMSE, Boston Naming Task, RAVLT, and the Color-Shape Task-switching Task were administered due to time constraints.

### Statistical Analysis

Separate analyses were done for each of the cognitive measures, i.e., MMSE, RAVLT, and Clock Drawing Test. We used R (R Core Team, 2021) for the data analysis, and the performance indices were fitted into linear mixed models using *lme4* (Bates et al., 2015) and *lmerTest* (Kuznetsova et al., 2017) packages. In each model, time (pretraining vs posttraining; within-subject variable), treatment (control vs SL vs DL; between-subject variable), and the interaction between the two variables were entered as fixed effects. The continuous variables, age and years of education, were *z*-transformed using the R function scale() and were included as control variables. Individual factors including subject ID, monthly household income, and testing mode (i.e., remotely or in-person) were included as random effects (see Author Note 3). The *p*-value of each fixed effect was obtained using the mixed() function in the *afex* (Singmann et al., 2016) package (see Toh et al., 2022). Significant main effects and interaction effects were further examined by conducting post hoc pairwise comparisons with the *emmean* (Lenth, 2022) package. Estimated marginal means (EMMs) and standard error (SE) will be reported as descriptive statistics when reporting the post hoc pairwise comparisons because they are the default outcomes from the *emmean* package. The analyses were done separately for the CH group and the CI group. We expect a significant interaction effect between Time and Treatment in the cognitive measures in both the CH and CI groups. The cognitive improvement from pretraining to posttraining is expected to be larger in or be observed only in, the DL group compared to the SL group and the control group.

## Results

### Cognitively Healthy Older Adults

#### Mini-Mental State Examination

The main effects of Time and Treatment, and the interaction effect between Time and Treatment were not significant for MMSE (*p*s > .061).

#### Clock Drawing Test

**Figure 3.**
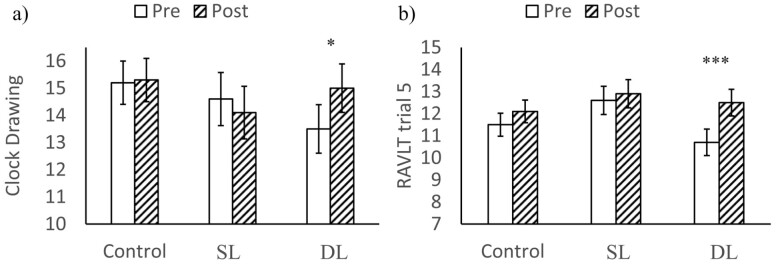
Comparisons between pretraining and posttraining by Treatment groups for (**a**) Clock Drawing Test score; and (**b**) RAVLT Trial 5 score in the cognitively healthy older adults. SL = single-language group; DL = dual-language group. Error bars indicate standard errors. Asterisks indicate significant differences between pretraining and posttraining. * *p* < .05. *** *p* < .001.

The main effects of Time and Treatment were not significant for the Clock Drawing Test (*p*s > .23), but the interaction effect of the two variables was significant (*χ*^*2*^(2)=6.20, *p* = .045) (see Figure 3a). Post hoc pairwise comparisons showed that the Clock Drawing Test scores were not significantly different between the pretraining and the posttraining for both the control group and the SL group (*p*s > .99), but the scores were significantly higher in the posttraining (EMM = 15.0, SE = 0.89) than in the pretraining (EMM = 13.5, SE = 0.89) for the ***DL group*** (*t*(43.1) = 2.75, *p* = .026, Cohen’s *d* = 0.57). The results indicated a significant positive effect of the DL cognitive training on the CH older adults’ visuospatial construction abilities.

#### Rey Auditory Verbal Learning Test (RAVLT)

The main effects of Treatment were not significant (*p*s > .112) in all RAVLT trials. Except for Trial 3 (*χ*^*2*^(1) = 0.26, *p* = .608), the main effects of Time in all RAVLT trials were significant (*p*s < .031) where participants’ posttraining scores generally improved significantly from pretraining scores.

The interaction effects of Time and Treatment were significant only in Trial 3 (*χ*^*2*^(2) = 6.99, *p* = .030) and Trial 5 (*χ*^*2*^(2) = 8.63, *p* = .013), but not in other trials (*p*s > .096). Post hoc pairwise comparisons between pre and posttraining in all three groups were not significant for Trial 3 (*p*s > .23), but were significant for Trial 5, where we found that the DL group improved significantly in Trial 5 score from pretraining (EMM = 10.7, SE = 0.60) to posttraining (EMM = 12.5, SE = 0.60; *t*(46) = 5.11, *p* < .001, Cohen’s *d* = 1.01), but not the control group or the SL group (*ps* >. 29) (see Figure 3b). Overall, results indicated a significant positive effect of the DL cognitive training on the CH older adults’ late verbal learning. Supplementary Material Section D summarizes the results related to the interaction effects of the above cognitive measures in CH older adults.

### Cognitively Impaired Older Adults

The main effects of Time and Treatment, and the interaction effects of the two variables were not significant (*ps* > .059) in MMSE, Clock Drawing Test, and all RAVLT trials. Supplementary Material Section D summarizes the results related to the interaction effects of the above cognitive measures in CI older adults.

#### Follow-up assessment

Twenty-five CI participants took part in the follow-up assessment. Two sets of linear mixed models were run. The construction of the linear mixed models was similar to those concerning the contrasts between the pretraining session and the posttraining session, except that the first set of models concerned the contrast between the pretraining session and the follow-up session so the Time variable is set to be pretraining versus follow-up, whereas the second set of models concerned the contrast between posttraining session and follow-up session, so the Time variable was set to be posttraining versus follow-up.

In the pretraining versus follow-up contrasts, the main effect of Time was significant in MMSE (*χ*^*2*^(1) = 7.81, *p* = .005) and RAVLT Trial 1 (*χ*^*2*^(1) = 5.30, *p* = .021). MMSE scores decreased from pretraining (EMM = 17.3, SE = 1.61) to follow-up session (EMM = 15.4, SE = 1.62; *t*(23.2) = 2.89, *p* = .008), and the RAVLT Trial 1 score improved from pretraining (EMM = 1.65, SE = 0.39) to follow-up session (EMM = 2.40, SE = 0.42; *t*(38.5) = 2.19, *p* = .035). The main effects of Time were not significant (*ps > *.40) in other RAVLT trials. Most importantly, the main effect of Treatment was significant in the delayed recall trial of RAVLT (*χ*^*2*^(2) = 7.29, *p* = .026). The ***DL group*** (EMM = 1.32, SE = 0.54) scored higher than the control group (EMM = 0.53, SE = 0.51; *t*(32.3) = 2.46, *p* = .058) in the delayed recall trial at a marginally significant level. SL group did not score significantly different from the DL group and control group (*ps* > .17) in the Delay Recall Trial. The main effects of Treatment, interaction effects of Time and Treatment were not significant (*ps* > .064) in all the other cognitive measures.

In the posttraining versus follow-up contrasts, the main effect of Time was significant in RAVLT Trial 1 (*χ*^*2*^(1) = 8.35, *p* = .004). RAVLT Trial 1 score improved from posttraining (EMM = 1.55, SE = 0.43) to follow-up session (EMM = 2.40, SE = 0.44). However, the main effects of Time, the main effects of Treatment, and the interaction effects of Time and Treatment were not significant (*ps* > .14) in all cognitive measures.

#### Exploratory pairwise comparison

**Figure 4.**
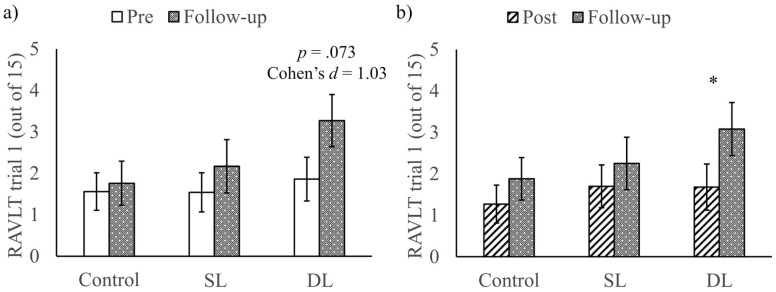
Comparisons between (**a**) pretraining and follow-up; and (**b**) posttraining and follow-up by Treatment groups for RAVLT trial 1 score. SL = single-language group; DL = dual-language group. Error bars indicate standard errors. The *p*-value and Cohen’s *d* indicate the significance and the effect size of the difference between pretraining and follow-up. Asterisks indicate significant differences between posttraining and follow-up. * *p* < .05.

The sample size of the CI participants returning for the follow-up session was small and might mask some potential results, thus, we conducted exploratory pairwise comparisons for each cognitive measure (for summary, see Supplementary Material Section E). In each exploratory pairwise comparison, the comparisons were Bonferroni-corrected. Exploratory pairwise comparisons revealed that the RAVLT trial 1 score was marginal significantly higher in the follow-up session (EMM = 3.27, SE = 0.63) than in the pretraining session (EMM = 1.86, SE = 0.53; *t*(35.6) = 2.35, *p* = .073) in the ***DL group*** (Figure 4a), and the effect size was large (Cohen’s *d* = 1.03). Similarly, the RAVLT trial 1 score was significantly higher in the follow-up session (EMM = 3.08, SE = 0.64) than in the posttraining session (EMM = 1.68, SE = 0.56; *t*(34) = 2.61, *p* = .040, Cohen’s *d* = 1.06) in the ***DL group*** (Figure 4b). The differences were not significant (*ps* > .58, Cohen’s *d*s < 0.464) in the control group and the SL group. The results indicated a possible delayed positive effect of the DL cognitive training on the CI older adults’ early verbal learning. No other exploratory pairwise comparisons were significant in other cognitive measures (*ps* > .145).

### Care Center Staff Feedback

On average, staff satisfaction towards the DISC was 4.13 out of 5 (SD = 0.64), which was significantly higher than the neutral rating of 3.0 (*t*(7) = 4.97, *p* = .002). Positive feedback related to the integration with center program included: the contents of DISC matched with some of the center programs, the older adults were interested in DISC, the staff agreed that playing DISC can improve the older adults’ cognitive skills, and the older adults could play DISC when they were bored with other games available in the centers. The staff rated a mean of 4.38 out of 5 (*SD* = 0.74) in agreeing that DISC benefits older adults and was significantly higher than the neutral rating (*t*(7) = 5.23, *p* = .001). These benefits included how older adults had proven to be able to learn how to use electronic devices, how DISC had generated interest in high-functioning dementia patients, how some of the older adults showed good memory, and how most of the older adults were happy to play DISC.

Six out of the eight staff (75%) either agreed that they had more time to focus on other clients or that they could perform more duties than they normally do when the researchers were running the DISC program. The two that disagreed were both counter staff of the centers so they were not involved in the daily care of the older adults. Among those who agreed, they indicated that running the DISC program had given them 5 to 60 minutes extra time to focus on other clients or perform more duties. The duties that they could perform included conducting assessments, running group activities, assisting other clients’ daily activities (e.g., toileting, feeding), having one-to-one coaching with other clients, working on documentation, and preparing food. The reasons for not having more time included that they needed to observe the participants’ behaviors and emotions and to ensure participants’ safety.

## Discussion

Our DISC project was the first to integrate the concept of DL context from the adaptive control hypothesis (Green & Abutalebi, 2013) into cognitive training developed for both CH and CI older adults. We hypothesized that the DL cognitive training of DISC would provide significant improvements in verbal learning and cognitive flexibility over SL cognitive training or no training (control group) in older adults. We observed significant posttraining improvements in the DL cognitive training group in the later stage of verbal learning (indexed by the RAVLT Trial 5 score) as well as visuospatial construction skills (indexed by the Clock Drawing Test score) in the CH older adults, and delayed improvement in early stage of verbal learning (indexed by the RAVLT Trial 1 score) in the CI older adults.

Our study contributed two important findings: (a) theoretical contributions of the role of bilingualism in older adults’ cognitive reserve, and (b) translational significance in the design and implementation of cognitive training programs. From the theoretical perspective, our findings support the cognitive adaptation facilitated by bilingualism as predicted by the adaptive control hypothesis (Green & Abutalebi, 2013), where DL context engages a higher demand for cognitive resources compared with the SL context. Such an increase in demand could contribute to the protective effects of lifelong bilingualism in neuroplasticity and cognitive reserve in older adults. Our results provided promising novel support for this hypothesis through systematically varying bilingual usage to examine its effects on cognitive functioning in older adults.

From the translation perspective, our study demonstrated a meaningful implementation of a novel language-based multi-modal touchscreen cognitive intervention tool based on established findings of cognitive benefits arising from a DL context. Our DISC program was designed to address some limitations observed in the traditional cognitive training programs, including labor intensiveness, dependence on caregiver/staff (e.g., see limitations in Kanaan et al., 2014), poor comprehension of instruction language (e.g., DeDe & Flax, 2016), and limited training effect size in cognitive domains, such as verbal memory, non-verbal memory, and visuospatial skills in cognitively healthy older adults (e.g., Lampit et al., 2014). First, to reduce the reliance on caregiver staff, improve instruction comprehension, and encourage independent gameplay from the older participants, we incorporated an interactive avatar in the game to provide audio instructions and to guide the users through the tutorials step-by-step. The users could also ask the avatar to repeat the instructions, with four different levels to cater to differing needs by tapping her if they had missed the instructions. There was a general reduction in the number of help requests during gameplay after we had revised the design of the prompt mechanism (Yow et al., 2020). From the qualitative feedback obtained after the intervention program, most of the care center staff agreed that having older adults play DISC did free up time for them to focus on other clients or perform other duties, from as little as 5 minutes to as long as an hour. Thus, our design allows older clients to understand our instructions easily and the care center staff to use the time to perform other duties instead of sitting next to the clients to guide them through the games. Secondly, in contrast to the generally small training effects of the traditional cognitive training programs (e.g., Lampit et al., 2014), the effect sizes of our findings ranged from medium (Cohen’s *d* = 0.57) to large (Cohen’s *d* = 1.01–1.06). Overall, our findings suggest that DL features with user-friendly elements can be incorporated into the design of cognitive training programs to enhance their effectiveness in mitigating cognitive decline in older adults.

We noted, however, that the effects of our DL cognitive training did not extend to nonverbal cognitive flexibility. It is possible that our training duration may have been too short to induce improvements in nonverbal cognitive abilities. Studies on children have shown that while DL education is associated with better verbal working memory and verbal learning skills than SL education in children in Kindergarten to 2nd Grade (Kaushanskaya et al., 2014), its benefits on nonverbal executive abilities (e.g., inhibition and switching) only emerged in 2nd Grade and 4th Grade (Esposito & Baker-Ward, 2013; Neveu et al., 2021). As our study only lasted for 2–3 months, the limited intervention duration could explain the absence of DL cognitive training-related improvement in nonverbal cognitive abilities. In addition, our training tasks focused on training verbal cognitive abilities rather than nonverbal cognitive abilities. Sala et al. (2019) suggested that there is no significant far transfer of working memory training, such that training on working memory does not improve trainees’ nonworking memory abilities such as fluid reasoning, cognitive control, processing speed, and language. Therefore, it is possible that training tasks that target nonverbal cognitive abilities have to be included to render similar benefits in these abilities.

Interestingly, while the DL training improved the later stage of verbal learning of the CH older adults immediately after the intervention, the beneficial effect of DL training on CI older adults’ early stage of verbal learning emerged only six months after the intervention was completed. One possible implication for this observation is that the effect of the DL training manifested differently between the CH and CI older adults. A number of neuroimaging studies have attempted to map the neural correspondences of each stage of verbal learning (e.g., each RAVLT trial) in older adults (e.g., Afthinos et al., 2022; Putcha et al., 2019). Past studies showed that the process of late verbal learning in older adults was uniquely associated with the left hippocampal volume and the perfusion at the angular gyrus, which are related to the conversion of short-term memory into long-term storage and semantic processing, respectively (Afthinos et al., 2022; Price, 2010; Putcha et al., 2019; Wang & Morris, 2010). Thus, DL training may bolster semantic encoding and memory consolidation in CH older adults. However, for early verbal learning, it is the cortical thickness of the dorsal attention network and the perfusion at the superior temporal gyrus (STG) that are commonly associated with the modulation of attention and phonetic encoding, respectively (Afthinos et al., 2022; Majerus et al., 2018; Mesgarani et al., 2014; Putcha et al., 2019). Therefore, it is possible that for CI older adults, the benefits of the DL training may be limited to the initial learning stages that require top-down attention regulation or phonetic feature encoding. This is also consistent with Putcha et al. (2019) where they found that MCI older adults showed greater impairment in the late verbal learning process than in the early verbal learning process.

We, however, do not have a strong explanation for the delayed cognitive improvement observed in the exploratory analyses for the CI older adults in the follow-up assessments. Similar delayed improvement has been observed in other cognitive training studies in older adults (e.g., Borella et al., 2017; Teixeira-Santos et al., 2022). The phenomenon has been termed as the “sleeper” effect, which describes a stronger intervention-related effect observed in the follow-up session than immediately after the intervention. The phenomenon is difficult to interpret, but some researchers speculate that the effect occurs because neural changes take a longer time to manifest at the behavioral level (Loewy et al., 2022). The phenomenon may be related to the neural degeneration experienced by older adults as suggested by Teixeira-Santos et al. (2022). It is nonetheless important to note that the number of CI older adults per treatment group who came back for the follow-up session was small, hence, we need to exercise caution when interpreting the findings from the follow-up session. Further longitudinal studies with neural measures and a larger sample size can be conducted to examine the possible “sleeper” effect of the cognitive benefits related to the DL cognitive training program in the CI populations.

CH older adults in the DL training condition showed improved visuospatial construction skills. This is in line with past studies that have found that bilingualism protects white matter integrity (e.g., Luk et al., 2011), which visuospatial abilities are associated with (Fan et al., 2019). Although to the best of our knowledge, there are no previous studies that examined the relationship between bilingualism and Clock Drawing Test scores, there are studies that have demonstrated other visuospatial benefits of bilingualism (e.g., Kerrigan et al., 2017; Luo et al., 2013). For instance, Luo et al. (2013) showed that visuospatial working memory, measured by the Corsi blocks test, was significantly better in bilinguals than in monolingual older adults. The bilingual advantage in the visuospatial memory was replicated by Kerrigan et al. (2017) using other visuospatial memory measures. Nevertheless, further studies should include more visuospatial measures to examine the effects of dual-language cognitive training on various aspects of visuospatial skills.

Although our study focused on the cognitive benefits of DL training and did not examine the clinical significance of the training, past studies showed that the Clock Drawing Test is one of the important indicators that predict the future dementia progression of a cognitively healthy older adult (Tsiaras et al., 2023; Umegaki et al., 2020), as well as whether an MCI older adult will revert back to healthy cognition in the near future (Tsiaras et al., 2023). Similarly, the RAVLT score is found to correlate with more independent activities of daily living in healthy older adults (Hartle et al., 2022; Mograbi et al., 2014) and better quality of life in Parkinson’s Disease patients (Olchik et al., 2016). Furthermore, the RAVLT score is suggested to predict whether an MCI patient would remain as MCI or progress to dementia within 2 years to a decade (Dawidowicz et al., 2021; Quaranta et al., 2023). Nevertheless, we should consider incorporating clinically relevant measures when evaluating the effectiveness of our program in the future.

There are limitations in our study. First, we did not have a large sample size, compounded by the difficulty of recruitment that spanned the COVID-19 pandemic. Additionally, for some tasks, there was missing data because some participants did not perform the task as instructed or because of administrative errors, which could have further reduced the power to detect small-to-medium effects. Second, due to the COVID-19 outbreak during our data collection period, some sessions were conducted online rather than in person. Although we tried to control for the effects of different experimental platforms in the data analysis, some noise could have been introduced due to the two different mediums of testing. Third, we acknowledge that we did not record the frequency of the control group participants in engaging each of the care center activities, such as playing bingo, reading newspapers, etc. It is recommended that future studies include measures on the frequency and engagement level in activities of the control participants. Fourth, we did not conduct any formal hearing or vision assessment on our participants, although older adults who were diagnosed with visual and auditory disabilities were not selected to participate in this study. We also did not observe any participants who showed sensory impairment during the intervention. Nevertheless, future studies should consider including hearing and visual tests of the participants. Lastly, as the study was conducted in Singapore, which is a multilingual country with multilingual citizens, further studies need to be conducted to examine if the same effects apply when compared with monolinguals in the single language condition or to other linguistically homogeneous versus diverse populations.

In conclusion, we developed a novel DL cognitive training program for older adults that showed positive results on verbal learning abilities and visuospatial construction skills among CH older adults. We also found a delayed improvement in early verbal learning skills after the CI older adults underwent DL cognitive training. Our study suggests a novel approach of using bilingualism as a cognitive intervention to mitigate cognitive decline in older adults, which is potentially scalable amongst bilingual populations for the greatest community impact.

## Supplementary Material

igae052_suppl_Supplementary_Materials

## Data Availability

Data reported in this article is available on request. The study was not preregistered.

## Author Notes

1. The overall score is 30. We used the education-adjusted cutoff scores recommended for multiethnic Asian populations (Chua et al., 2019). A score of 19 or below is classified as cognitively impaired for individuals whose highest education level is primary school, and 23 or below for secondary school graduates.

2. DISC has been renamed as Ami (**A**dvancing **M**ental **I**nvigoration) app at the launch of our prototype app at the Lions Befrienders Service Association (Singapore) in September 2023.

3. The full model: Cognitive measures ~ Condition*Test + Age + Years of Education + (1|Participant ID) + (1|Sex) + (1|Income) + (1|Remote:Participant ID)
