# Supplementary material for: A Novel Dual-Language Touch-Screen Intervention to Slow Down Cognitive Decline in Older Adults: A Randomized Controlled Trial
Source: Innov Aging. 2024 May 27;8(7):igae052. doi: 10.1093/geroni/igae052 (PMC11227001; doi:10.1093/geroni/igae052)
Supplement: igae052_suppl_Supplementary_Materials [file igae052_suppl_supplementary_materials.docx]

***Innovation in Aging* Supplementary Material: Yow et al. A Novel Dual-Language Touch-Screen Intervention to Slow Down Cognitive Decline in Older Adults – A Randomized Controlled Trial.**

**Section A**

**Table A1.** Design of Object Categorization, Verbal Fluency, and Utility of Things

| **Task** | **Object Categorization** | **Verbal fluency** | **Utility of Things** |
| --- | --- | --- | --- |
| **Baseline Measures** | Press 12 Stars on the screen | 1. List out things in the room  2. Name the things on your body | Select the target circle out of two presented circles |
| **Practice Trials** | Themes:   1. Fruits 2. Furniture | Themes:   1. Flowers 2. Transportation | Themes:   1. It can be eaten 2. It can be used for cutting |
| **Actual Trials** | 6 themes, 4 choices each | 6 themes, 45 Seconds for each | 6 themes, 4 choices each (total of 24 trials) |
| **Themes** | Animals | Animal | It can be eaten |
|  | Fruits | Fruits | It can be worn |
|  | Vegetables | Vegetables | It can be used for reading |
|  | Shapes | Supermarket items | It can be used for writing |
|  | Furniture | Bedroom items | It can be used for cooking |
|  | Colors | Colors | It can be used for cutting |
| *Notes.* The order of the themes for the actual trials of the three game tasks, and for the practice trials of the Verbal Fluency task and the Utility of Things task are randomized. | | | |

**Section B**

**Game Design**

***Game Tasks***

All three game tasks are developed based on past studies that used similar intervention tasks (Brewer et al., 1981, and Flicker et al., 1987 for Object Categorization; Monsch et al., 1992 for Verbal Fluency task; Tárraga et al., 2006 for Utility of Thing task). In the Object Categorization task, participants are to choose four out of twelve items based on a given category. The correct images would move into the squares on the right of the screen once selected. In the Verbal Fluency task, participants are to verbally name as many items as they can within 45 seconds that belong to a given category. A star would appear once the participants had completed a trial. Lastly, in the Utility of Things task, participants are to choose one of two objects that best fits an aforementioned function (e.g. an object that can be used for eating).

***Avatar***

DISC features an avatar called Ah Mei (a familiar local name in Singapore) that delivers instructions, feedback upon completion of tasks, and prompts if participants face any difficulties while playing the game. The avatar is dressed in a uniform that is similar to what care center staff would typically wear in Singapore and speaks in a local accent. The name and outfit were used to create a sense of familiarity between participants and the character, which aims to increase the participants’ motivation and interest in the app. Instructions used colloquial phrases for ease of understanding. Ah Mei delivers instructions and comments such as “Well done!”, “That’s not right”, and “Please try again!” in a positive tone. Most of the instructions and audio feedback are supported by visual elements, such as arrows, ticks, and stars for further emphasis.

Participants can touch the avatar for help where needed. With each touch, the avatar would voice prompts of four different levels, with prompt 1 giving the simplest level of help (e.g., repeating the instructions) and prompt 4 giving the most help (e.g., removing all the incorrect options). To assist task completion among older adults, DISC incorporates accessibility features such as enlarged timer and images, a teal-colored background, and the ability to accommodate several types of touch interactions (e.g., long taps, double taps, and tapping while instructions are playing).

***Language Features***

There are two language modes available in DISC – single-language (SL) mode and dual-language (DL) mode. On the login page of DISC, the participant can choose the first language and the second language they would like to play the game in. If the participant chooses the same language for the first language and the second language, the game will be played in SL mode. The four language options are English, Mandarin, Cantonese, and Hokkien, which are commonly used languages among older adults in Singapore in daycare centers.

In the SL mode, all the task instructions are delivered in the single language that the participant has chosen to play in. In the DL mode, the task instructions alternate between two languages in a single trial (e.g., the instruction in the SL mode could be: “Now the theme is “Fruits”. Let’s select the pictures from this category. For example, the apple is a fruit, so we can select it.” In the DL mode, the same instruction would be: “Now the theme is “Fruits”. Let’s select the pictures from this category. 比如, 苹果是一种水果, 请你按下苹果的图片.”). Specifically for the Verbal Fluency task, participants respond either in one language (SL mode) or in alternate languages (e.g., cat, 狗，rat, 象; DL mode). The design of the DL mode is modeled after the DL context in bilingual communication that is believed to render the most cognitive demands as per the adaptive control hypothesis (Green & Abutalebi, 2013).

**Section C**

**Boston Naming Test**

A modified local version of the Boston Naming Test was used (Lee et al., 2012). Thirty pictures were presented individually, either on an A4-sized piece of white paper or as a slide presentation on a 13-inch laptop. Participants were given 30 seconds to name each picture. No feedback was given. 1 point was given for each question answered correctly. Thus, the range of possible scores for the Boston Naming Test was 0 to 30.

**Digit Symbol Substitution Test (DSST)**

The DSST is part of the Wechsler Adult Intelligence Scale (Wechsler, 1955) and is a neuropsychological test that can be used to measure cognitive deficits (Jaeger, 2018). This test was administered via paper and pencil. Participants were required to fill in a series of symbols that corresponded to numbers 1-9 within 90 seconds. It requires response speed, sustained attention, visuospatial skills, and set shifting. There were 10 practice items before 90 test trials started. 1 point was given for each item answered correctly. Thus, the range of possible scores for the DSST was 0 to 90.

**Color-Shape Task-Switching Task**

The Color-Shape Task-Switching Task was adapted from the study by Prior and Macwhinney (2010). In this task, participants were instructed to categorize a target stimulus either by its color (i.e., red or green) or by its shape (i.e., triangle or square), according to the task cue presented prior to the target. The color task cue was a color gradient strip, and the shape task cue was an array of white dots. Participants were instructed to press the “O” key if the target was red or triangle, and the “P” key if the target was green or square.

Participants completed a practice block, followed by two single-cue blocks and then four mixed-cue blocks. Each single-cue block contained only one type of task cue (i.e., *single-task trials*), and participants completed a block of color-task trials and a block of shape-task trials, with the order counterbalanced across participants. Half of the trials in the mixed-cue block were *non-switch trials*, where the task of the current trial was the same as that of the previous trial (e.g., color-color), and the other half were *switch trials*, in which the task of the current trial was different from that of the previous trial (e.g., color-shape) (see Figure A1).

Mixing cost and switching cost were calculated based on the RTs in the single-cue blocks and the mixed-cue blocks. Switching cost is a measure of more transient control processes related to task changing across trials while mixing cost reflects proactive control mechanisms maintaining two competing task goals (see Li et al., 2021; Rubin & Meiran, 2005). The larger the values of mixing cost and switching cost, the weaker the cognitive flexibility.


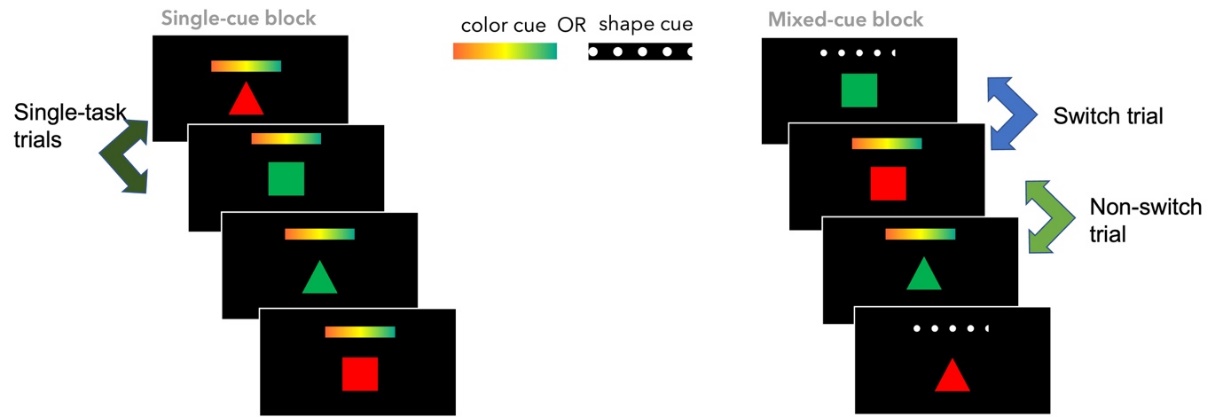


**Figure A1.** Examples of the designs of the single-cue block and the mixed-cue block of the Color-Shape Task-Switching Task.

**N-back Task**

N-back task is used to assess working memory, with multiple layers of cognitive processes, and has different levels of difficulty. There were three levels: 0-back (Figure A2a), 1-back (Figure A2b), and 2-back (Figure A2c). In the 0-back task, participants were presented with a series of targets and were tasked to press the spacebar if an “X” appeared on the screen. In the 1-back task and the 2-back task, participants were presented with a series of digits and were tasked to press the spacebar if the current digit matched the digit one trial (for the 1-back task) or two trials (for the 2-back task) before. The performances of the N-back tasks were indexed by d’.


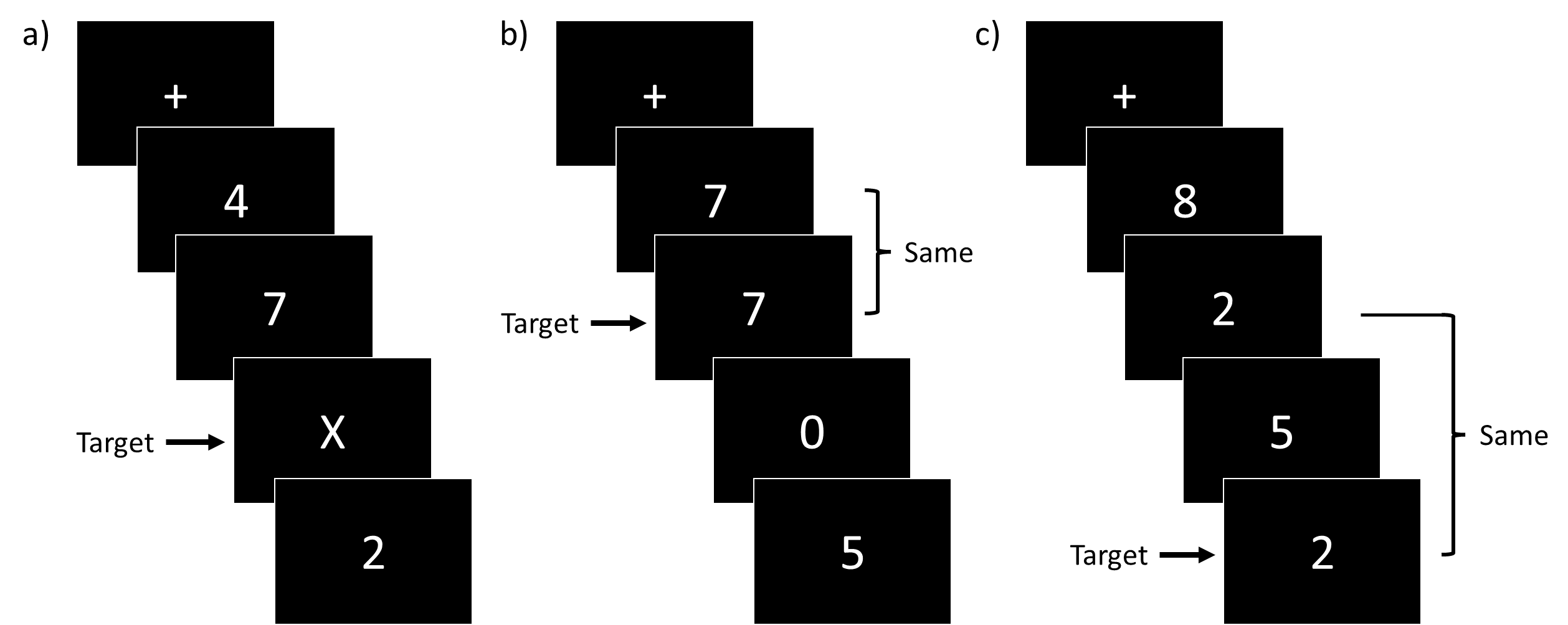


**Figure A2**. Example of a) the 0-back task; b) the 1-back task; and c) the 2-back task.

**Section D**

**Table A2.** Summary of the interaction effects between Time and Treatment of cognitively healthy participants and cognitively impaired participants.

|  | *χ^2^* | *p* |
| --- | --- | --- |
| Cognitively healthy participants | | |
| MMSE | 5.59 | .061 |
| Clock Drawing Test | 6.20 | .045 |
| RAVLT Trial 1 | 3.94 | .139 |
| RAVLT Trial 2 | 4.68 | .096 |
| RAVLT Trial 3 | 6.99 | .030 |
| RAVLT Trial 4 | 0.19 | .911 |
| RAVLT Trial 5 | 8.63 | .013 |
| RAVLT Delayed Recall | 3.93 | .140 |
| Cognitively impaired participants | | |
| MMSE | 0.34 | .844 |
| Clock Drawing Test | 0.47 | .789 |
| RAVLT Trial 1 | 0.49 | .781 |
| RAVLT Trial 2 | 0.12 | .941 |
| RAVLT Trial 3 | 1.54 | .462 |
| RAVLT Trial 4 | 0.44 | .803 |
| RAVLT Trial 5 | 0.95 | .621 |
| RAVLT Delayed Recall | 0.62 | .735 |
| *Note.* MMSE, Mini-Mental State Examination. RAVLT, Rey Auditory Verbal Learning Test. | | |

**Section E**

**Table A3.** Summary of the exploratory pairwise comparison for the follow-up contrasts in the cognitively impaired participants.

|  | **Group** | **Pretraining**  **EMM (SE)** | **Follow-up**  **EMM (SE)** | ***p*** | **Cohen’s *d*** |
| --- | --- | --- | --- | --- | --- |
| Pretraining – Follow-up contrast | | | | | |
| MMSE | DL | 17.0 (2.33) | 14.6 (2.43) | .145 | 0.48 |
|  | SL | 17.4 (1.59) | 15.1 (2.06) | .229 | 0.46 |
|  | Control | 17.5 (1.67) | 16.6 (1.76) | >.999 | 0.17 |
| RAVLT Trial 1 | DL | 1.86 (0.53) | 3.27 (0.63) | .073 | 1.03 |
|  | SL | 1.54 (0.47) | 2.17 (0.64) | >.999 | 0.46 |
|  | Control | 1.56 (0.45) | 1.76 (0.53) | >.999 | 0.14 |
| RAVLT Trial 2 | DL | 2.88 (0.70) | 3.56 (0.83) | >.999 | 0.40 |
|  | SL | 2.69 (0.59) | 3.02 (0.76) | >.999 | 0.20 |
|  | Control | 2.43 (0.52) | 2.45 (0.63) | >.999 | 0.01 |
| RAVLT Trial 3 | DL | 3.65 (0.74) | 4.39 (0.84) | .847 | 0.44 |
|  | SL | 2.98 (0.62) | 3.24 (0.76) | >.999 | 0.16 |
|  | Control | 3.16 (0.54) | 2.90 (0.63) | >.999 | 0.15 |
| RAVLT Trial 4 | DL | 3.64 (0.79) | 3.85 (0.85) | >.999 | 0.12 |
|  | SL | 3.38 (0.66) | 3.17 (0.74) | >.999 | 0.12 |
|  | Control | 3.26 (0.57) | 3.05 (0.63) | >.999 | 0.12 |
| RAVLT Trial 5 | DL | 3.93 (0.79) | 3.84 (0.86) | >.999 | 0.05 |
|  | SL | 3.96 (0.66) | 3.70 (0.77) | >.999 | 0.14 |
|  | Control | 3.36 (0.59) | 2.95 (0.65) | >.999 | 0.23 |
| RAVLT Delayed Recall | DL | 0.94 (0.55) | 1.69 (0.60) | .189 | 0.62 |
|  | SL | 0.83 (0.53) | 0.31 (0.61) | .728 | 0.43 |
|  | Control | 0.55 (0.52) | 0.50 (0.56) | >.999 | 0.04 |

|  | **Group** | **Posttraining**  **EMM (SE)** | **Follow-up**  **EMM (SE)** | ***p*** | **Cohen’s *d*** |
| --- | --- | --- | --- | --- | --- |
| Posttraining – Follow-up contrast | | | | | |
| MMSE | DL | 16.9 (2.02) | 15.5 (2.17) | .769 | 0.31 |
|  | SL | 16.9 (1.75) | 16.4 (1.90) | >.999 | 0.11 |
|  | Control | 16.3 (1.54) | 16.2 (1.59) | >.999 | 0.02 |
| RAVLT Trial 1 | DL | 1.68 (0.56) | 3.08 (0.64) | .040 | 1.06 |
|  | SL | 1.70 (0.52) | 2.25 (0.63) | >.999 | 0.41 |
|  | Control | 1.27 (0.46) | 1.88 (0.51) | .581 | 0.46 |
| RAVLT Trial 2 | DL | 2.60 (0.77) | 3.15 (0.87) | >.999 | 0.31 |
|  | SL | 2.75 (0.67) | 3.29 (0.78) | >.999 | 0.30 |
|  | Control | 2.22 (0.58) | 2.52 (0.65) | >.999 | 0.17 |
| RAVLT Trial 3 | DL | 3.91 (0.76) | 4.16 (0.89) | >.999 | 0.14 |
|  | SL | 3.40 (0.66) | 3.46 (0.80) | >.999 | 0.03 |
|  | Control | 2.86 (0.57) | 2.80 (0.66) | >.999 | 0.03 |
| RAVLT Trial 4 | DL | 3.67 (0.88) | 3.68 (0.96) | >.999 | 0.01 |
|  | SL | 3.88 (0.76) | 3.31 (0.85) | >.999 | 0.29 |
|  | Control | 3.66 (0.65) | 2.90 (0.71) | .388 | 0.38 |
| RAVLT Trial 5 | DL | 4.77 (0.81) | 3.69 (0.95) | .462 | 0.56 |
|  | SL | 4.18 (0.71) | 3.75 (0.86) | >.999 | 0.22 |
|  | Control | 3.44 (0.61) | 3.01 (0.71) | >.999 | 0.23 |
| RAVLT Delayed Recall | DL | 0.39 (0.68) | 1.03 (0.75) | .529 | 0.42 |
|  | SL | 0.98 (0.58) | 0.55 (0.65) | >.999 | 0.28 |
|  | Control | 0.21 (0.51) | 0.30 (0.54) | >.999 | 0.06 |
| *Note.* MMSE, Mini-Mental State Examination. RAVLT, Rey Auditory Verbal Learning Test. DL, dual-language group. SL, single-language group. EMM, estimated marginal mean. SE, standard error. The *p*-values are Bonferroni-corrected for multiple comparison. | | | | | |

**References**

Brewer, M. B., Dull, V., & Lui, L. (1981). Perceptions of the elderly: Stereotypes as prototypes. *Journal of Personality and Social Psychology, 41*(4), 656–670.

Flicker, C., Ferris, S. H., Crook, T., & Bartus, R. T. (1987). Implications of memory and language dysfunction in the naming deficit of senile dementia. *Brain and Language, 31*(2), 187–200.

Green, D. W., & Abutalebi, J. (2013). Language control in bilinguals: The adaptive control hypothesis. *Journal of Cognitive Psychology, 25*(5), 515–530. https://doi.org/10.1080/20445911.2013.796377

Jaeger, J. (2018). Digit Symbol Substitution Test. *Journal of Clinical Psychopharmacology, 38*(5), 513–519. https://doi.org/10.1097/JCP.0000000000000941

Lee, C. K. Y., Collinson, S. L., Feng, L., & Ng, T.-P. (2012). Preliminary Normative Neuropsychological Data for an Elderly Chinese Population. *The Clinical Neuropsychologist, 26*(2), 321–334. https://doi.org/10.1080/13854046.2011.652180

Li, X., Ng, K. K., Wong, J. J. Y., Lee, J. W., Zhou, J. H., & Yow, W. Q. (2021). Bilingual language entropy influences executive functions through functional connectivity and signal variability. *Brain and Language, 222*, 105026. https://doi.org/10.1016/j.bandl.2021.105026

Monsch, A. U., Bondi, M. W., Butters, N., Salmon, D. P., Katzman, R., & Thal, L. J. (1992). Comparisons of Verbal Fluency Tasks in the Detection of Dementia of the Alzheimer Type. *Archives of Neurology, 49*(12), 1253–1258. https://doi.org/10.1001/archneur.1992.00530360051017

Prior, A., & Macwhinney, B. (2010). A bilingual advantage in task switching. *Bilingualism: Language and Cognition, 13*(2), 253–262. https://doi.org/10.1017/S1366728909990526

Rubin, O., & Meiran, N. (2005). On the Origins of the Task Mixing Cost in the Cuing Task-Switching Paradigm. *Journal of Experimental Psychology: Learning, Memory, and Cognition, 31*(6), 1477–1491. https://doi.org/10.1037/0278-7393.31.6.1477

Tárraga, L., Boada, M., Modinos, G., Espinosa, A., Diego, S., Morera, A., Guitart, M., Balcells, J., López, O. L., & Becker, J. T. (2006). A randomised pilot study to assess the efficacy of an interactive, multimedia tool of cognitive stimulation in Alzheimer’s disease. *Journal of Neurology, Neurosurgery, and Psychiatry, 77*(10), 1116–1121. https://doi.org/10.1136/jnnp.2005.086074

Wechsler, D. (1955). *Manual for the Weschsler Adults Intelligence Scale (WAIS).* The Psychological Corporation.
